# Diabetes duration or age at onset and mortality in insulin-dependent diabetics: a systematic review and meta-analysis

**DOI:** 10.1186/s13098-023-01113-x

**Published:** 2023-07-01

**Authors:** Xing-mu Wang, Shu-ping Zhong, Gang-feng Li, Fu-yuan Zhuge

**Affiliations:** 1 Clinical Laboratory Center, Shaoxing People’s Hospital (Shaoxing Hospital, Zhejiang University School of Medicine), Shaoxing, Zhejiang People’s Republic of China; 2 Department of Hospital Management, Shaoxing People’s Hospital (Shaoxing Hospital, Zhejiang University School of Medicine), Shaoxing, Zhejiang People’s Republic of China; 3 Department of Endocrine and Metabolism, Shaoxing People’s Hospital (Shaoxing Hospital, Zhejiang University School of Medicine), Yuecheng District, No.568, Zhongxing North Road, Shaoxing, Zhejiang People’s Republic of China

**Keywords:** Diabetes duration, Age at onset, IDDM, Mortality, Risk, Systematic review, Meta-analysis

## Abstract

**Background:**

This meta-analysis was conducted given the contradictory findings from studies on the influence of diabetes duration or age at onset on mortality in patients with insulin-dependent diabetes mellitus (IDDM).

**Methods:**

Electronic databases (PubMed, Embase, Cochrane, Web of Knowledge, Scopus, and CINHAL) were comprehensively searched to identify relevant studies until October 31, 2022. All of the selected articles contained statistics on hazard ratios, relative risks (RRs), or odds ratios, or data for estimating the association between diabetes duration or age at onset and total mortality in IDDM patients. Regardless the heterogeneity assessed by the I^2^ statistic, pooled RRs and 95% confidence intervals (CI) for total mortality were acquired via random effect meta-analysis with inverse variance weighting.

**Results:**

This meta-analysis finally included 19 studies involving 122, 842 individuals. Both age at onset and diabetes duration were positively associated with an increased mortality rate in IDDM patients. Specifically, the pooled RRs for age at onset and diabetes duration were 1.89 (95%CI 1.43–2.50) and 1.89 (95%CI 1.16–3.09) respectively. Subgroup analyses revealed that only prepubertal onset was associated with a greater survival advantage than pubertal or postpubertal onset.

**Conclusions:**

The findings of this meta-analysis and systematic review suggest that a later age at onset or longer diabetes duration is associated with increased risk of total mortality in IDDM patients. However, this conclusion shall be interpreted with caution due to the possibility of residual confounding and be confirmed in the future by well-designed studies.

**Supplementary Information:**

The online version contains supplementary material available at 10.1186/s13098-023-01113-x.

## Background

Diabetes mellitus (DM) is a serious public global health concern. More than half a billion people (536.6 million) aged 20–79 years were expected to develop DM in 2021, with about 90% having noninsulin-dependent diabetes mellitus (NIDDM) [1]. In recent decades, diabetes-related health and economic burdens have increased globally, especially in low- and middle-income countries [2]. Most studies estimating diabetes burdens have focused on NIDDM, but paid scant attention to insulin-dependent diabetes mellitus (IDDM) [3]. As a significant chronic autoimmune disease, IDDM affects adolescents and children frequently [4], and becomes more prevalent worldwide in the last decade [5]. Since 1989, the global incidence of IDDM in children under 14 has increased by 3% annually [6], and about 8.4 million people worldwide would have IDDM in 2021 [7]. Misdiagnosis, underdiagnosis, a considerable chance of complications, and premature mortality are obstacles [8, 9].

Evidence from the past confirms that age at onset is closely related to premature mortality among IDDM patients [10]. According to a Swedish study, it discovered that patients who developed IDDM before the age of 10 years, and 26—30 years had a threefold, and less than twofold increase in mortality respectively compared to controls [11]. A Finnish study determined that the standardised mortality ratio for the early onset (0–14 years) cohort was 3.6 and that for the late onset (15–29 years) cohort was 2.80 [12]. Likewise, a recent narrative review found a correlation between the onset age of IDDM and total mortality [13]. This review had numerous limitations, including the absence of subgroup analysis and failure to recognize heterogeneity. Specifically, the omission of certain confounders (e.g., study design, early-onset criteria, and model adjustment ignorance) may result in bias. In addition, a previous analysis of 13 population-based EURODIAB registers from 12 countries found inconsistent results and no significant difference in the standardised mortality ratios by age at diagnosis [14].

In terms of diabetes duration, a longer duration implies an earlier onset, and a lower mortality risk shall be anticipated. This is not the situation, however. No systematic review or meta-analysis of the relationship between diabetes duration and mortality in diabetic populations has been conducted to date. In light of these considerations, our objective is to determine if diabetes duration or age at onset influences the total mortality of diabetic patients.

## Methods

### Search strategy and inclusion criteria

PubMed EMBASE, Wiley Cochrane Central Register of Controlled Trials (CENTRAL), Web of Science, Scopus, and CINAHL were exhaustively searched until October 31, 2022 in accordance with the Preferred Reporting Items for Systematic Reviews and Meta-Analyses [15]. The search terms included "diagnostic age" or "diagnosis age" or "age of diagnosis" or "age on diagnosis" or "age at diagnosis" or "age at onset" or "onset age" or "age of onset" or "childhood onset" or "adolescent onset" or "puberty onset" or "prepuberty onset" or "duration of diabetes" or "time of diabetes" or "period of diabetes" (search strategy shown in Additional file 1). The references in pertinent articles were manually looked through to identify potential articles. WD and YZ searched independently, and any disagreement between them was coped by team consensus. The authors were contacted to obtain required information. If a site-specific dataset had been previously published, the most recent publication was chosen.

The inclusion criteria were: (i) observational study (cohort or case–control); (ii) reporting the relationship of diabetes duration or age at onset (age at diagnosis) with total mortality in IDDM patients; (iii) reporting effect estimates: standardized mortality or incidence ratio (SMR or SIR), or hazard ratio (HR), relative risk (RR) or odds ratios (OR), and relevant raw data for re-calculation. The exclusion criteria were: (I) case report, quasiexperiment (non-random subject allocation), editorial, remark, review, or unpublished study; (II) only published as abstract or conference proceeding. Studies that did not report such estimates for IDDM but also included T2D were excluded.

### Data extraction and quality evaluation

Information regarding the study, participants (mean attained and current age, and gender), analysis strategy (statistical models, adjustment factors), and effect magnitude (e.g., SMR or IRR, or HRs, RRs or ORs) was gathered along with relevant raw data for recalculation. Specifically, the study information included design, name of first author, title, publication year, country/region, calendar time period of study, follow-up time, endpoints, sample size, adjustment level, measure of association, numbers of observed and expected events. The study quality was evaluated separately by WD and YZ using the 9-star Newcastle–Ottawa Scale (NOS). A rating greater than six stars indicates high quality [16]. Disagreements were handled by discussion.

### Statistical analysis

The principal result measure was total mortality. As a control for the age at onset, the earliest age at onset or prepubertal onset was used. The associations between age groups and total mortality in the same study, relative to the controls, were merged before being combined with measurements from other studies. In terms of diabetes duration, only the shortest duration was used as a control, and the associations of different age groups, relative to the controls, with total mortality from the same study were combined before being combined with measurements from other studies.

The percent of between-study variability attributable to between-study heterogeneity was estimated by the I^2^ statistic [17] and categorized as high, modest or low with I^2^ ≥ 50%, < 50% and < 25%, respectively. The iterative non-central Chi-2 method [18] was used to identify a CI for I2.

Regarding the a-priori discrepancy of OSs, we performed subgroup analyses for the association between age at onset and total mortality according to sample size (< 1000, and ≥ 1000), control group (prepubertal, and others), age at onset/age at diagnosis, and type of effect measure (reported and calculated). Identifiable sources of dissimilarity were clarified by removing the articles one by one in a sensitivity test.

When 5 or more studies were available for analysis, Begg’s and Egger’s tests were used to test publication bias, and the dissymmetry of funnel plots of estimated effects versus standard errors was visually inspected [19]. Duval & Tweedie’s trim-and-fill method was used to correct for any publication bias (P < 0.10). All other analyses were performed on STATA 12.0 (US) at the significance level of P < 0.05.

## Results

### Study identification and quality evaluation

**Fig.1:**
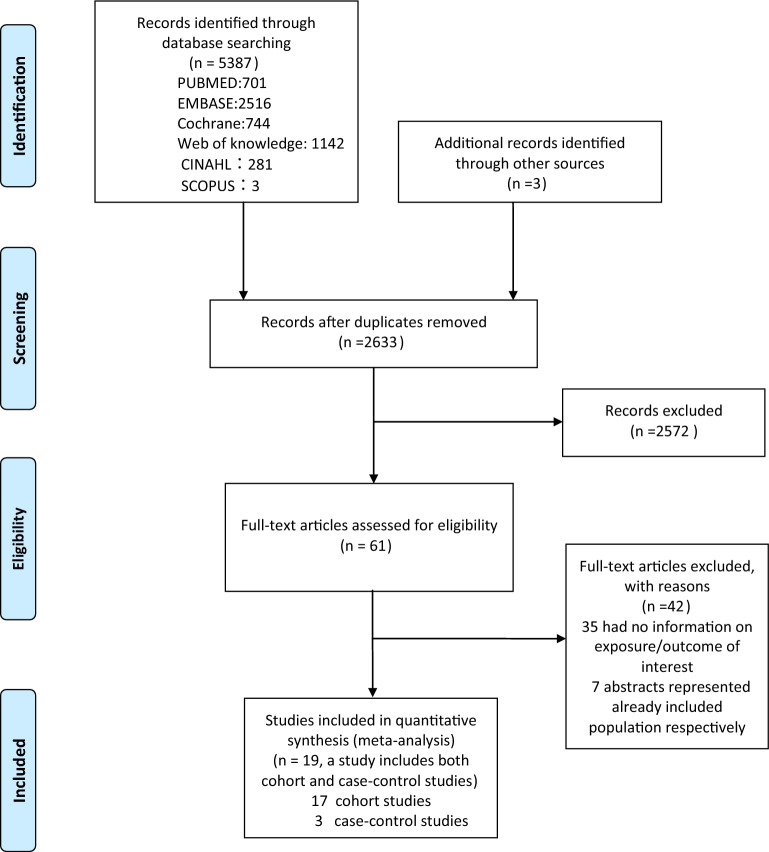
Flow-diagram of study selection

The systematic search yielded 5,390 publications, of which 61 were chosen for additional review (Fig. 1). Two articles [20, 21] from the same data source presented contradictory findings, and thus were both included. Two studies [22, 23] from the same team or institution with different results were included for analysis. Two studies [24, 25] from the same team or institution both reported an independent variable with age at onset and age at diagnosis and thus were both included. Finally, 20 papers provided information on the link between diabetes duration or age at onset and total death (Table 1). The 20 studies included two case–control studies [10, 26], 17 cohort studies [11, 20–23, 25, 27–37], and one study [24] that combined both case–control and cohort studies. The sample sizes of these studies ranged from 103 to 27,195 patients, and the follow-up periods ranged from 3.0 to 33.0 years. The share increased from 40.05% to 72.22%, while diabetes duration increased from 3.5 to 31.9 years.

**Table 1:** Detailed characteristics of studies included in the meta-analysis

Study	Data source/Country/Region	Study design	Calendar time period of study	Age at onset/Age at diagnosis/diabetes duration	Sample size	Mean attained age/Mean current age (years)	Female (%total)	Mean diabetes duration(Years)	Mean follow-up time(Years)	Effect estimate of all-cause mortality
Kostraba et al.(1991)	Children's Hospital of Pittsburgh Insulindependent Diabetes Mellitus Registry of/USA	Case–control	1950–1981	Age at onset	924	28.32	87.88	19.1	20.0	RR
Modan et al.(1991)	the DERI study group activitie/Israel	Cohort	1965–1979	Age at diagnosis	614	NR	NR	NR	11.5	OR(calc)
COLLADO-MESA et al.(1997)	Havana City Province data from the National Registry of IDDM/Cuba	Cohort	1965–1980	Age at diagnosis	504	NR	48.6	25.0	17.5	HR(extracting from Kapla-Meier curves)
NlSHIMURA et al.(1998)	two nationwide IDDM surveys/Japan	Case–control Cohort	1965–1979	Age at onset	1,286	23.28	40.05	NR	11.55	Case–control: RR Cohort: OR(calc)
Mühlhause et al. (2000)	the diabetes centre of the Düsseldorfuniversity Hospital/Germany	Cohort	1978–1994	Diabetes duration	3,570	27.5	50.3	10.6	10.3	HR
ASAO et al.(2003)	Nationwide surveys/Japan; the National Social Insurance Institution /Finland	Cohort	1965–1979	Age at diagnosis	Japan:1,408 Finland:5,126	NR	Japan:59.80 Finland:45.04	Japan:3.5 Finland: 4.2	Japan: 16.3 Finland:17.8	Japan: aHR Finland: aHR
Barceló et al.(2007)	Havana City Province data from the National Registry of IDDM/Cuba	Cohort	AC:1965–1979 HA:1965–1980	Age at onset	504	NR	48.61	16.5	NR	OR(calc)
Rendas-Baum et al. (2006)	the New Jersey 725 study/USA	Case–control	1993–1998	Diabetes duration	725	29.00	58.34	9.50	3.0	HR
Dawson et al.(2008)	the Canterbury Diabetes Registry/ New Zealand	Cohort	1984–2004	Age at onset	989	52.36	50.66	16.80	13.60	OR(calc)
SECREST et al.(2010)	The Allegheny County Type 1 Diabetes Registry cohort/ USA	Cohort	1965–1979	Age at onset	1,075	42.8	48.0	31.9	33.0	OR(calc)
Washington et al.(2014)	the USVI Childhood (< 19 years old) Diabetes Registry/USA	Cohort	1979–2005	Age at onset	103	28.2	52.4	16.8	16.8	OR(calc)
Gagnum et al.(2015)	the nationwide, population based Norwegian Childhood Diabetes Registry/Norway	Cohort	1973–1982; 1989–2012	Age at diagnosis	7,884	NR	46.0	16.8	16.8	aHR
Marshall et al.(2016)	Rwanda Life For a Child (LFAC) program/USA、 Australia、Rwanda	Cohort	2004–2012	Age at diagnosis	488	20.95	57.38	4.5	NR	aHR
Cheung et al.(2017)	YRDCYP/United Kingdom	Cohort	1978–2013	Age at diagnosis	5,498	NR	45.96	NR	17.42	aHR
Gomes et al. (2017)	BNHCS /Brazil	Cohort	2014–2015	Age at diagnosis/diabetes duration	986	28.5	54.4	15.9	16.1	OR(calc)
Rawshani et al. (2018)	the Swedish National Diabetes Register/Sweden	Cohort	1998–2012	Age at onset	27,195	NR	44.2	13.0	10.0	OR(calc)
Conway et al. (2018)	the Southern Community Cohort Study/USA	Cohort	2002–2009	Age at diagnosis	475	49.87	66.32	29.06	9.5	OR(calc)
Groop et al. (2018)	FinnDiane/Finland	Cohort	1980–2005	Age at onset	10,737	35.51	45.83	16.2	14.0	OR(calc)
Majaliwa et al. (2022)	CYLDM/ Tanzania	Cohort	1991–2004; 2005–2010; 2011–2019	Age at diagnosis	3,235	NR	49.2	5.0	NR	OR(calc)

*BNHCS* the Brazilian National Health Care System, *DERI* Diabetes Epidemiology Research International, *YRDCYP* The Yorkshire Register of Diabetes in Children and Young People, *AC* Allegheny County, *HA* Havana, *USVI* U.S. Virgin Islands, *FinnDiane* the Finnish Diabetic Nephropathy Study, *CYLDM* Children and Youth Living with Diabetes Mellitus, *IDDM* Insulin dependent diabetes mellitus, *aHR* Adjusted Hazard Ratio, *RR* Relative Risk/Risk Ratio, *OR* Odds Ratio, *calc* Calculate, *NR* not reported

Aside from the multinational origins in one study [33], other origins included developed countries or regions in 15 articles [10, 11, 22–32, 35, 36], and developing countries in only four articles [20, 21, 34, 37]. Three articles [22, 26, 28] and sixteen studies [10, 11, 20, 21, 23–25, 27, 29–33, 35–37] presented data comparing diabetes duration and age at onset, respectively, with the risk of total mortality, and one study [34] presented data comparing either diabetes duration or age at onset with total mortality.

With ≥ 7 NOS scores (mean = 7.5; Additional file 2: Table S2), all 20 OSs were of high methodological quality.

### Impact of diabetes duration on total mortality in IDDM patients

**Fig. 2:**
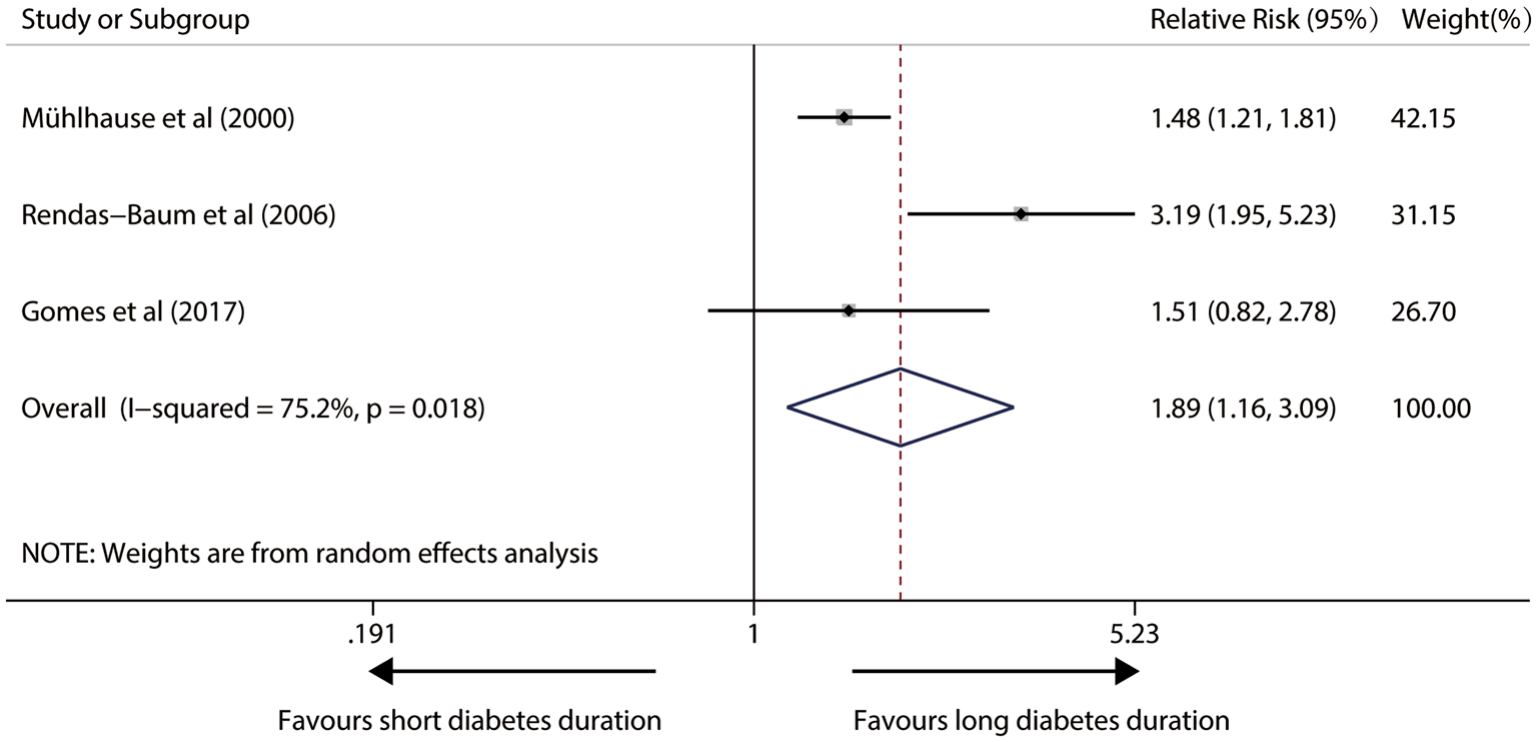
Forest plot for the association between diabetes duration and risk of total mortality in IDDM patients (The X-axis represents the log scale; the solid square represents the relative risk; and the horizontal lines represent the 95% CIs. The same in other figures)

In a random-effects model with significant heterogeneity (I^2^ = 75.2%; P = 0.018) from four relevant studies, patients with longer diabetes duration had a 89% higher risk (pooled RR, 1.89; 95%CI 1.16–3.09; P = 0.011) in susceptibility to any death. Figure 2 illustrates forest plots of the meta-analysis.

### Impact of age at onset on total mortality in IDDM patients

**Fig. 3:**
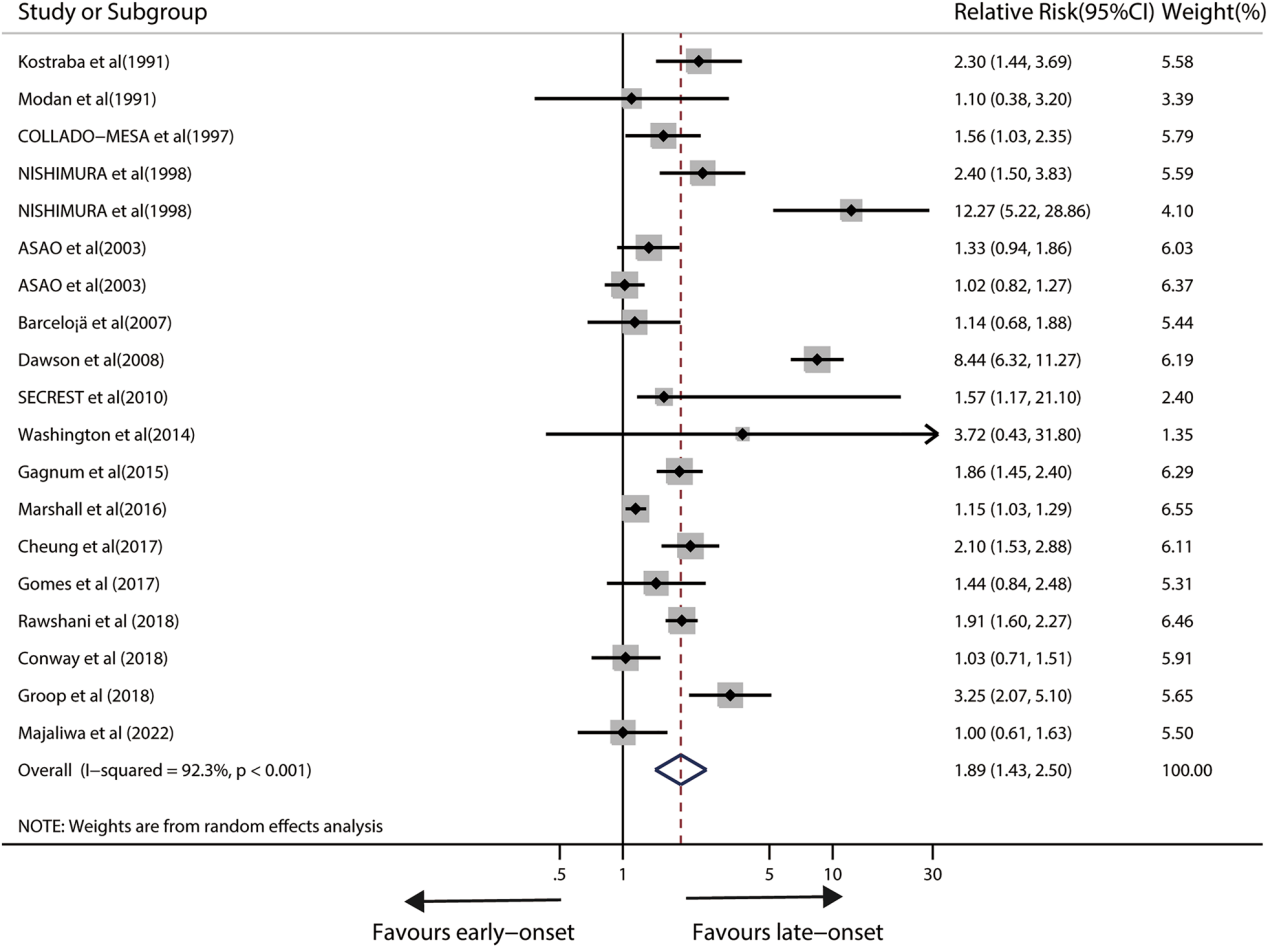
Forest plot for the association between age at onset and risk of total mortality in IDDM patients

Seventeen relevant studies were included in the analysis of age at onset/age at diagnosis with the danger of total mortality. Overall, the pooled RR showed a 89% greater risk of total death in late-onset TID patients compared to early-onset IDDM patients (RR,1.89; 95%CI 1.43–2.50; p < 0.001), however, there was evident heterogeneity among trials (I^2^ = 92.3%, p < 0.001) (Fig. 3). After removing single studies, sensitivity analysis showed that heterogeneity did not disappear. Neither Egger's test (P = 0.183) nor visual check revealed any substantial publication bias (Additional file 3: Fig.S1).

**Fig. 4:**
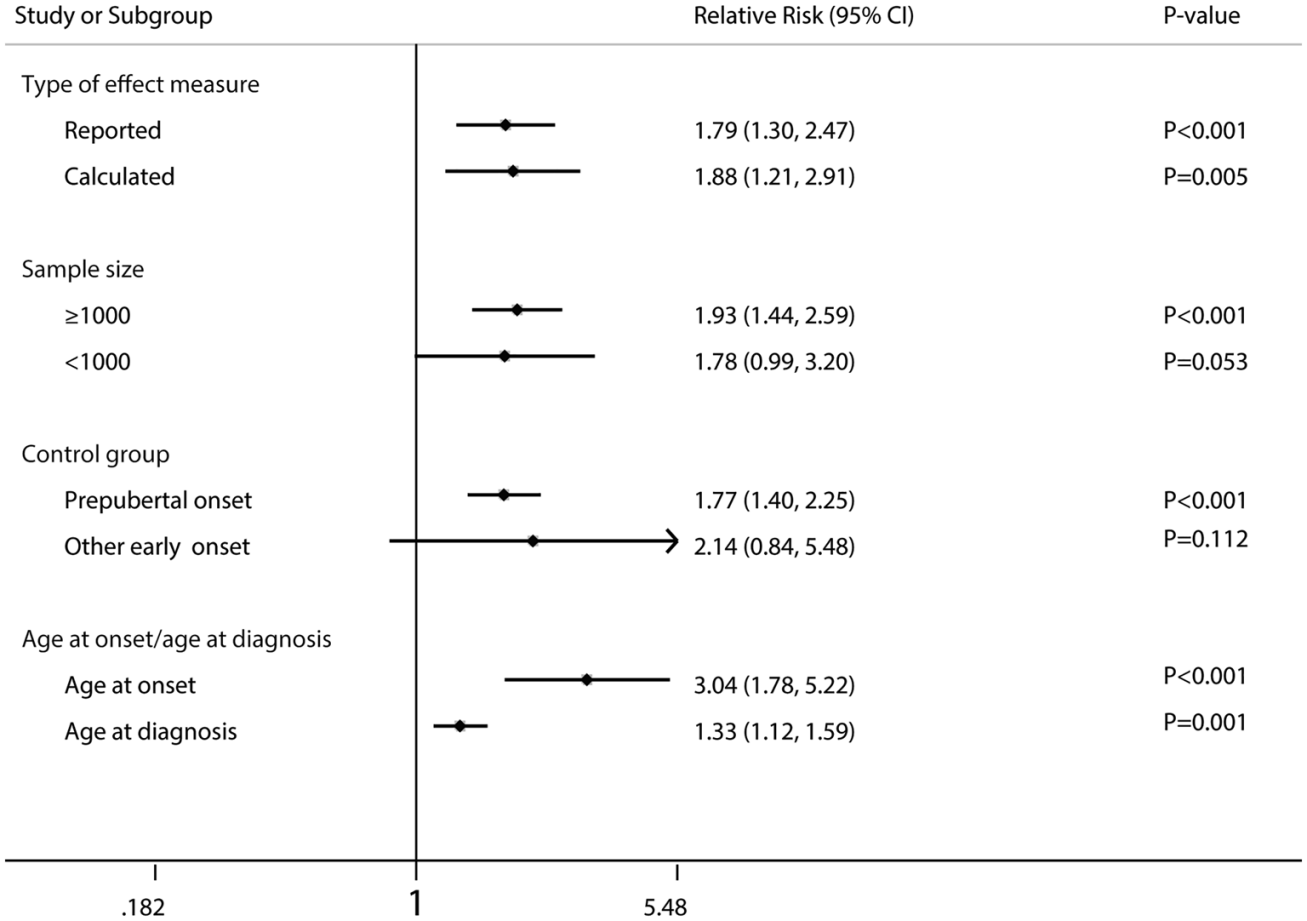
Forest plot for the association between age at onset and risk of total mortality in IDDM patients according to some clinically important variables. *IDDM* insulin-dependent diabetes mellitus

The pooled RRs were broadly consistent across the large sample size (≥ 1000 patients, P < 0.001), age at onset/age at diagnosis (both P ≤ 0.001), prepubertal onset as control group (P < 0.001), and type of effect measure (reported, P < 0.001; calculated, P = 0.005) (Fig. 4).

## Discussion

We are the first to comprehensively examine and meta-analyze the discrepancy between early-late onset or diabetes duration and total mortality in IDDM patients. Our findings reveal that IDDM people have a greater risk of mortality over longer time period with diabetes. In regard to early-late onset, we searched all current studies involving 69,031 people and discovered a significantly higher risk of total mortality for those with a later onset than those with an earlier onset. Only prepubertal onset offered a survival advantage according to further subgroup analyses. Furthermore, age at onset and age at diagnosis were both positively connected with the risk of total mortality, with age at onset being more evenly distributed. Although the underlying biological mechanisms directly linking prepubertal onset or diabetes duration to death have yet to be determined, there are three accepted hypotheses. (1) As reported [38], diabetes duration has been linked to the prevalence and prognosis of diabetic complications, and the impact of age at onset on mortality may be attributed to a differential effect of puberty on duration in the etiology of microvascular complications [10]. It is further proposed that the age at onset is marked significantly by duration and not by age, because the persistence of diabetes after puberty will predict mortality, regardless of age. (2) The etiology of IDDM is heterogeneous, with a more benign disease diagnosed before the age of 12 years, and the diabetic complications progress due to a different pathogenesis [10, 39–41] and worse glycemic control. This is so due to the impaired insulin action [42] caused by increased secretion of various hormones during puberty [43–45], by psychosocial issues [46], and differences in self-management education and experience, additional to differences in shifting health services from pediatric to adult populations [35]. (3) In the peripubertal group, the duration from onset to diagnosis may be longer than in the younger population, contributing to a longer period of potential organ damage [20].

Notably, Araz Rawshani et al. [11] discovered that patients who developed IDDM between the age of 0 and 10 had hazard ratios for all-cause mortality of 4.11 in contrast to the general population, after adjusting for duration. They believed that diabetes duration was significant since total correction was unattainable. Diabetes duration is a component of total glycemic load. Glycaemic load is defined as the cumulative exposure of the vasculature to glucose and is affected by diabetes duration and glycaemic variability. A larger glycemic burden and hence the damage lead to longer duration of diabetes (similar to the area under the LDL cholesterol exposure curve) [47, 48]. Obviously, coronary arteries are particularly sensitive to hyperglycemia, and possibly even more when hyperglycemia first manifests during the first 10 to 15 years of life. Another possible explanation for their findings is that individuals with a younger age of onset have a more severe and rapid loss of β-cells due to a distinct type of insulitis [49, 50], which contributes to elevated glycemia. Furthermore, after 10 years of disease duration, children and teenagers with IDDM begin to suffer subclinical cardiovascular disease abnormalities, as exemplified by numerous methodologies [51–53]. Given these contradicting reports, more research is needed to determine whether such disparities among age-at-onset groups are connected to a different etiology of disease based on age at onset.

Using raw data from Araz Rawshani's et al. study, we discovered that prepubertal onset was preferable to later onset. The explanation is unknown, but can be due to an ambiguous achieved age, as there is considerable debate about whether diabetes duration or age of onset is the key predictor of increased relative mortality. This was because the pubertal group had diabetes for a shorter time period than the prepubertal group at a given attained age. A given attained age simultaneously represents background risks. Studies [25, 32, 54, 55] found that attained age, rather than diabetes duration or age at diagnosis, was the most crucial predictor of outcome. Because attained age is the sum of age at onset and duration of diabetes, it cannot be assessed in a single multivariate analysis model. Reportedly, diabetes duration had a strong but variable impact on relative mortality, and age at diagnosis, in conjunction with diabetes duration, was the most crucial predictor [55]. None of the parameters influenced the relative mortality of patients with a transient diabetes history. Age at initiation and diabetes duration at admission were the primary indicators of higher relative mortality in diabetic individuals with median duration. However, after 40 years, almost all of the factors had lost their significance. One study [23] also revealed that duration of diabetes did not impact diabetic complications as severely as previously thought.

The detailed retrieval plan utilizing Cochrane protocols and the relatively large sample size are two significant strengths of this meta-analysis. This study has some limitations. First, the avoidance of unpublished reports may have skewed our results. Second, the onset age and diagnosis of IDDM cannot be discriminated precisely, and the misclassification may obscure a significant link. Third, significant heterogeneity was discovered due to the lack of uniformity in the case elucidation method, study design and period, endpoint classification, and the adjusted level of between-study confounding. These results need be confirmed by other research because we cannot take into account the vast majority of between-study heterogeneity, despite the sensitivity testing. Fourth, no information was available on the treatment or other clinical factors (e.g., HbA1c level). In consequence, it is challenging to identify the factors that influence how mortality risk changes. Fifth, studies with smaller sample sizes (N < 1000) were included in the meta-analysis, but these studies very probably lacked the statistical power to identify the true association.

## Conclusion

In summary, our study provides further evidence for the association between age at onset or diabetes duration, and mortality risk in IDDM patients. However, this conclusion must be understood carefully given the potential remaining confounding factors and be further validated by well-planned prospective studies.

## Supplementary Information

**Additional file 1: Table S1.** Search stragely.

**Additional file 2: Table S2.** Quality assessment of observational studies included in the meta-analysis by NOS. *NOS* Newcastle–Ottawa scale.

**Additional file 3: Fig. S1.** Funnel plot for the association between age at onset and risk of total mortality in IDDM patients. *IDDM* insulin-dependent diabetes mellitus.

## Data Availability

The datasets used and analyzed in this investigation are accessible from the corresponding author in response to a legitimate request.
