# Supplementary material for: Diabetes duration or age at onset and mortality in insulin-dependent diabetics: a systematic review and meta-analysis
Source: Diabetol Metab Syndr. 2023 Jul 1;15:147. doi: 10.1186/s13098-023-01113-x (PMC10314605; doi:10.1186/s13098-023-01113-x)
Supplement: Supplementary file 1 — Additional file 1: Table S1. Search stragely. [file 13098_2023_1113_MOESM1_ESM.docx]

**Search stragely**

**Pubmed**

#1 “[diagnostic](https://wenda.so.com/q/1412029104726533) age”[Title/Abstract] OR “diagnosis age”[Title/Abstract] OR “age of diagnosis”[Title/Abstract] OR “age on diagnosis”[Title/Abstract] OR “age at diagnosis”[Title/Abstract] OR “early diagnosis”[Title/Abstract] OR “Childhood diagnosis” [Title/Abstract] OR “Adolescent diagnosis” [Title/Abstract] OR “[puberty](https://fanyi.so.com/?src=onebox#puberty) diagnosis” [Title/Abstract] OR “pre[puberty](https://fanyi.so.com/?src=onebox#puberty) diagnosis” [Title/Abstract] OR “late diagnosis”[Title/Abstract] OR “young diagnosis”[Title/Abstract] OR “old diagnosis”[Title/Abstract]

#2 “onset age”[Title/Abstract] OR “age of onset”[Title/Abstract] OR “Childhood onset”[Title/Abstract] OR “adolescent onset”[Title/Abstract] OR “[puberty](https://fanyi.so.com/?src=onebox#puberty) onset”[Title/Abstract] OR “pre[puberty](https://fanyi.so.com/?src=onebox#puberty) onset”[Title/Abstract] OR “late onset”[Title/Abstract] OR “young onset”[Title/Abstract] OR “old onset”[Title/Abstract] OR “first episode”[Title/Abstract] OR “age at onset”[Title/Abstract]

#3 “duration of diabetes”[Title/Abstract] OR “Time of diabetes” [Title/Abstract] OR “Length of diabetes” [Title/Abstract] OR “Period of diabetes” [Title/Abstract] OR “span of diabetes” [Title/Abstract] OR “year of diabetes” [Title/Abstract] OR “years of diabetes” [Title/Abstract] “Diabetes duration”[Title/Abstract] OR “Diabetes time”[Title/Abstract] OR “Diabetes length”[Title/Abstract] OR “Diabetes span”[Title/Abstract] OR “Diabetes year”[Title/Abstract] OR “Diabetes years”[Title/Abstract]

#4 #1 OR #2 OR #3

#5 "Diabetes mellitus, type 1"[MeSH Terms] OR "Diabetic Ketoacidosis"[MeSH Terms] OR "Diabetes Complications"[MeSH Terms]

#6 IDDM[Title/Abstract] OR T1DM[Title/Abstract] OR T1D[Title/Abstract] OR “typ* 1 diabet*”[Title/Abstract] OR “typ* I diabet*”[Title/Abstract] OR “insulin* depend*”[Title/Abstract] OR “insulin?depend*”[Title/Abstract] OR “earl* diabet*”[Title/Abstract] OR “auto?immun* diabet*”[Title/Abstract] OR “sudden onset diabet*”[Title/Abstract] OR “insulin* defic* absolut*”[Title/Abstract] OR “acidos* diabet”[Title/Abstract] OR “juvenil* diabet*”[Title/Abstract] OR “child* diabet*”[Title/Abstract] OR “keto* diabet*”[Title/Abstract] OR “labil* diabet”*[Title/Abstract] OR “britt* diabet*”[Title/Abstract]

#7 #5 OR #6

#8 “death”[Title/Abstract] OR “decease”[Title/Abstract] OR “demise”[Title/Abstract] OR “die”[Title/Abstract] OR “dead”[Title/Abstract] OR “loss of life”[Title/Abstract] OR “life time”[Title/Abstract] OR “lifetime”[Title/Abstract] OR “life span”[Title/Abstract] OR “duration of life”[Title/Abstract] OR “mortality”[Title/Abstract] OR “mortalit*”[Title/Abstract] OR “length of life”[Title/Abstract] OR “life duration” [Title/Abstract] OR “longevity”[Title/Abstract] OR “natural life”[Title/Abstract] OR “operating life”[Title/Abstract] OR “survival”[Title/Abstract]

#9 “death”[MeSH Terms]

#10 "[Mortality](https://www.ncbi.nlm.nih.gov/mesh/68009026)"[MeSH Terms]

#11 #8 OR #9 OR #10

#11 (animal[mh] not human[mh])

#12 comment[Publication Type] OR editorial[Publication Type] OR letter[Publication Type]

#13 #4 AND #7 AND #11 NOT#12 NOT #13

**Embase**

#1 ‘[diagnostic](https://wenda.so.com/q/1412029104726533) age’:ab,ti OR ‘diagnosis age’:ab,ti OR ‘age of diagnosis’:ab,ti OR ‘age on diagnosis’:ab,ti OR ‘age at diagnosis’:ab,ti OR ‘early diagnosis’:ab,ti OR ‘Childhood diagnosis’:ab,ti OR ‘Adolescent diagnosis’:ab,ti OR ‘[puberty](https://fanyi.so.com/?src=onebox#puberty) diagnosis’:ab,ti OR ‘pre[puberty](https://fanyi.so.com/?src=onebox#puberty) diagnosis’:ab,ti OR ‘late diagnosis’:ab,ti OR ‘young diagnosis’:ab,ti OR ‘old diagnosis’:ab,ti

#2 ‘onset age’:ab,ti OR ‘age of onset’:ab,ti OR ‘Childhood onset’:ab,ti OR ‘adolescent onset’:ab,ti OR ‘[puberty](https://fanyi.so.com/?src=onebox#puberty) onset’:ab,ti OR ‘pre[puberty](https://fanyi.so.com/?src=onebox#puberty) onset’:ab,ti OR ‘late onset’:ab,ti OR ‘young onset’:ab,ti OR ‘old onset’:ab,ti OR ‘first episode’:ab,ti OR ‘age at onset’:ab,ti

#3 ‘duration of diabetes’:ab,ti OR ‘Time of diabetes’:ab,ti OR ‘Length of diabetes’:ab,ti OR ‘Period of diabetes’:ab,ti OR ‘span of diabetes’:ab,ti OR ‘year of diabetes’:ab,ti OR ‘years of diabetes’:ab,ti OR ‘Diabetes duration’:ab,ti OR ‘Diabetes time’:ab,ti OR ‘Diabetes length’:ab,ti OR ‘Diabetes span’:ab,ti OR ‘Diabetes year’:ab,ti OR ‘Diabetes years’:ab,ti

#4 #1 OR #2 OR #3

#5 ‘insulin dependent diabetes mellitus’/exp OR ‘Diabetic Ketoacidosis’/exp OR ‘diabetic complication’/exp

#6 IDDM:ab,ti OR T1DM:ab,ti OR T1D:ab,ti OR “typ$ 1 diabet$”:ab,ti OR “typ$ I diabet$”:ab,ti OR “insulin$ depend$”:ab,ti OR “insulin?depend$”:ab,ti OR “earl$ diabet$”:ab,ti OR “auto?immun$ diabet$”:ab,ti OR “sudden onset diabet$”:ab,ti OR “insulin$ defic$ absolut$”:ab,ti OR “acidos$ diabet”:ab,ti OR “juvenil$ diabet$”:ab,ti OR “child$ diabet$”:ab,ti OR “keto$ diabet$”:ab,ti OR “labil$ diabet$”:ab,ti OR “britt$ diabet$”:ab,ti

#7 #5 OR #6

#8 “death”:ab,ti OR “decease”:ab,ti OR “demise”:ab,ti OR “die”:ab,ti OR “dead”:ab,ti OR “loss of life”:ab,ti OR “life time”:ab,ti OR “lifetime”:ab,ti OR “life span”:ab,ti OR “duration of life”:ab,ti OR “mortality”:ab,ti OR “mortalit$”:ab,ti OR “length of life”:ab,ti OR “life duration”:ab,ti OR “longevity”:ab,ti OR “natural life”:ab,ti OR “operating life”:ab,ti OR “survival”:ab,ti

#9 ‘death’/exp OR ‘mortality’/exp

#10 #8 OR #9

#11 #4 AND #7 AND #10

Lim #11 to human

**Cochrane**

#1 ([diagnostic](https://wenda.so.com/q/1412029104726533) age):ti,ab,kw OR (diagnosis age):ti,ab,kw OR (age of diagnosis):ti,ab,kw OR (age on diagnosis):ti,ab,kw OR (age at diagnosis):ti,ab,kw OR (early diagnosis):ti,ab,kw OR (Childhood diagnosis):ti,ab,kw OR (Adolescent diagnosis):ti,ab,kw OR ([puberty](https://fanyi.so.com/?src=onebox#puberty) diagnosis):ti,ab,kw OR (pre[puberty](https://fanyi.so.com/?src=onebox#puberty) diagnosis):ti,ab,kw OR (late diagnosis):ti,ab,kw OR (young diagnosis):ti,ab,kw OR (old diagnosis):ti,ab,kw

#2 (onset age):ti,ab,kw OR (age of onset):ti,ab,kw OR (Childhood onset):ti,ab,kw OR (adolescent onset):ti,ab,kw OR ([puberty](https://fanyi.so.com/?src=onebox#puberty) onset):ti,ab,kw OR (pre[puberty](https://fanyi.so.com/?src=onebox#puberty) onset):ti,ab,kw OR (late onset):ti,ab,kw OR (young onset):ti,ab,kw OR (old onset):ti,ab,kw OR (first episode):ti,ab,kw OR (age at onset):ti,ab,kw

#3 (duration of diabetes):ti,ab,kw OR (Time of diabetes):ti,ab,kw OR (Length of diabetes):ti,ab,kw OR (Period of diabetes):ti,ab,kw OR (span of diabetes):ti,ab,kw OR (year of diabetes):ti,ab,kw OR (years of diabetes):ti,ab,kw (Diabetes duration):ti,ab,kw OR (Diabetes time):ti,ab,kw OR (Diabetes length):ti,ab,kw OR (Diabetes span):ti,ab,kw OR (Diabetes year):ti,ab,kw OR (Diabetes years):ti,ab,kw

#4 #1 OR #2 OR #3

#5 MeSH descriptor:[Diabetes mellitus, type 1] explode all trees

#6 MeSH descriptor: [Diabetic Ketoacidosis] explode all trees

#7 MeSH descriptor:[Diabetes Complications] explode all trees

#8 IDDM:ti,ab,kw OR T1DM:ti,ab,kw OR T1D:ti,ab,kw OR (typ* 1 diabet*):ti,ab,kw OR (typ* I diabet*):ti,ab,kw OR (insulin* depend*):ti,ab,kw OR (insulin?depend*):ti,ab,kw OR (earl* diabet*):ti,ab,kw OR (auto?immun* diabet*):ti,ab,kw OR (sudden onset diabet*):ti,ab,kw OR (insulin* defic* absolut*):ti,ab,kw OR (acidos* diabet):ti,ab,kw OR (juvenil* diabet*):ti,ab,kw OR (child* diabet*):ti,ab,kw OR (keto* diabet*):ti,ab,kw OR (labil* diabet)*:ti,ab,kw OR (britt* diabet*):ti,ab,kw

#9 #5 OR #6 OR #7 OR #8

#10 (death):ti,ab,kw OR (decease):ti,ab,kw OR (demise):ti,ab,kw OR (die):ti,ab,kw OR (dead):ti,ab,kw OR (loss of life):ti,ab,kw OR (life time):ti,ab,kw OR (lifetime):ti,ab,kw OR (life span):ti,ab,kw OR (duration of life):ti,ab,kw OR (mortality):ti,ab,kw OR (mortalit*):ti,ab,kw OR (length of life):ti,ab,kw OR (life duration):ti,ab,kw OR (longevity):ti,ab,kw OR (natural life):ti,ab,kw OR (operating life):ti,ab,kw OR (survival):ti,ab,kw

#11 MeSH descriptor:[death] explode all trees

#12 #10 OR #11

#13 #4 AND #7 AND #12

#14 Limit #13 to clinical trails

**CINAHL (EBSCOhost)**

S1 TX(“[diagnostic](https://wenda.so.com/q/1412029104726533) age” OR “diagnosis age” OR “age of diagnosis” OR “age on diagnosis” OR “age at diagnosis” OR “early diagnosis” OR “Childhood diagnosis” OR “Adolescent diagnosis” OR “[puberty](https://fanyi.so.com/?src=onebox#puberty) diagnosis” OR “pre[puberty](https://fanyi.so.com/?src=onebox#puberty) diagnosis” OR “late diagnosis” OR “young diagnosis” OR “old diagnosis”)

S2 TX(“onset age” OR “age of onset” OR “Childhood onset” OR “adolescent onset” OR “[puberty](https://fanyi.so.com/?src=onebox#puberty) onset” OR “pre[puberty](https://fanyi.so.com/?src=onebox#puberty) onset” OR “late onset” OR “young onset” OR “old onset” OR “first episode” OR “age at onset” )

S3 TX(“duration of diabetes” OR “Time of diabetes” OR “Length of diabetes” OR “Period of diabetes” OR “span of diabetes” OR “year of diabetes” OR “years of diabetes” OR “Diabetes duration” OR “Diabetes time” OR “Diabetes length” OR “Diabetes span” OR “Diabetes year” OR “Diabetes years”)

S4 OR/S1-S3

S5 (MH"Diabetes mellitus, type 1")

S6 (MH"Diabetic Ketoacidosis")

S7 TI(IDDM OR T1DM OR T1D OR “typ* 1 diabet*” OR “typ* I diabet*” OR “insulin* depend*” OR “insulin?depend*” OR “earl* diabet*” OR “auto?immun* diabet*” OR “sudden onset diabet*” OR “insulin* defic* absolut*” OR “acidos* diabet” OR “juvenil* diabet*” OR “child* diabet*” OR “keto* diabet*” OR “labil* diabet”* OR “britt* diabet*”)

S8 AB(IDDM OR T1DM OR T1D OR “typ* 1 diabet*” OR “typ* I diabet*” OR “insulin* depend*” OR “insulin?depend*” OR “earl* diabet*” OR “auto?immun* diabet*” OR “sudden onset diabet*” OR “insulin* defic* absolut*” OR “acidos* diabet” OR “juvenil* diabet*” OR “child* diabet*” OR “keto* diabet*” OR “labil* diabet”* OR “britt* diabet*”)

S9 OR/S5-S8

S10 (MH"death")

S11 (MH"mortality")

S12 TI(“death” OR "decease" OR “demise” OR “die” OR “dead” OR “loss of life” OR “life time” OR “lifetime” OR “life span” OR “duration of life” OR “mortality” OR “mortalit*” OR “length of life” OR “life duration” OR “longevity” OR “natural life” OR “operating life” OR “survival”)

S13 AB(“death” OR "decease" OR “demise” OR “die” OR “dead” OR “loss of life” OR “life time” OR “lifetime” OR “life span” OR “duration of life” OR “mortality” OR “mortalit*” OR “length of life” OR “life duration” OR “longevity” OR “natural life” OR “operating life” OR “survival”)

S14 [S10](http://www.ncbi.nlm.nih.gov/pubmed/advanced) OR S11 OR [S12](http://www.ncbi.nlm.nih.gov/pubmed/advanced) OR S13

S15 S4 AND S9 AND S14

S16 limited S15 to human

**Web of knowledge**

[#1](http://www.ncbi.nlm.nih.gov/pubmed/advanced) TS=(“[diagnostic](https://wenda.so.com/q/1412029104726533) age” OR “diagnosis age” OR “age of diagnosis” OR “age on diagnosis” OR “age at diagnosis” OR “early diagnosis” OR “Childhood diagnosis” OR “Adolescent diagnosis” OR “[puberty](https://fanyi.so.com/?src=onebox#puberty) diagnosis” OR “pre[puberty](https://fanyi.so.com/?src=onebox#puberty) diagnosis” OR “late diagnosis” OR “young diagnosis” OR “old diagnosis” OR “onset age” OR “age of onset” OR “Childhood onset” OR “adolescent onset” OR “[puberty](https://fanyi.so.com/?src=onebox#puberty) onset” OR “pre[puberty](https://fanyi.so.com/?src=onebox#puberty) onset” OR “late onset” OR “young onset” OR “old onset” OR “first episode” OR “age at onset” OR “duration of diabetes” OR “Time of diabetes” OR “Length of diabetes” OR “Period of diabetes” OR “span of diabetes” OR “year of diabetes” OR “years of diabetes” OR “Diabetes duration” OR “Diabetes time” OR “Diabetes length” OR “Diabetes span” OR “Diabetes year” OR “Diabetes years”)(title, abstract, keywords)

[#2](http://www.ncbi.nlm.nih.gov/pubmed/advanced) TS=(IDDM OR T1DM OR T1D OR “typ* 1 diabet*” OR “typ* I diabet*” OR “insulin* depend*” OR “insulin?depend*” OR “earl* diabet*” OR “auto?immun* diabet*” OR “sudden onset diabet*” OR “insulin* defic* absolut*” OR “acidos* diabet” OR “juvenil* diabet*” OR “child* diabet*” OR “keto* diabet*” OR “labil* diabet”* OR “britt* diabet*”)(title, abstract, keywords)

[#3](http://www.ncbi.nlm.nih.gov/pubmed/advanced) TS=(“death” OR "decease" OR “demise” OR “die” OR “dead” OR “loss of life” OR “life time” OR “lifetime” OR “life span” OR “duration of life” OR “mortality” OR “mortalit*” OR “length of life” OR “life duration” OR “longevity” OR “natural life” OR “operating life” OR “survival”)

[#4](http://www.ncbi.nlm.nih.gov/pubmed/advanced) [#1](http://www.ncbi.nlm.nih.gov/pubmed/advanced) AND [#4](http://www.ncbi.nlm.nih.gov/pubmed/advanced) AND [#3](http://www.ncbi.nlm.nih.gov/pubmed/advanced)

**SCOPUS**

((“[diagnostic](https://wenda.so.com/q/1412029104726533) age” OR “diagnosis age” OR “age of diagnosis” OR “age on diagnosis” OR “age at diagnosis” OR “early diagnosis” OR “Childhood diagnosis” OR “Adolescent diagnosis” OR “[puberty](https://fanyi.so.com/?src=onebox#puberty) diagnosis” OR “pre[puberty](https://fanyi.so.com/?src=onebox#puberty) diagnosis” OR “late diagnosis” OR “young diagnosis” OR “old diagnosis”)(title, abstract, keywords) OR (“onset age” OR “age of onset” OR “Childhood onset” OR “adolescent onset” OR “[puberty](https://fanyi.so.com/?src=onebox#puberty) onset” OR “pre[puberty](https://fanyi.so.com/?src=onebox#puberty) onset” OR “late onset” OR “young onset” OR “old onset” OR “first episode” OR “age at onset”)(title, abstract, keywords) OR (“duration of diabetes” OR “Time of diabetes” OR “Length of diabetes” OR “Period of diabetes” OR “span of diabetes” OR “year of diabetes” OR “years of diabetes” OR “Diabetes duration” OR “Diabetes time” OR “Diabetes length” OR “Diabetes span” OR “Diabetes year” OR “Diabetes years”) (title, abstract, keywords)) AND (IDDM OR T1DM OR T1D OR “typ* 1 diabet*” OR “typ* I diabet*” OR “insulin* depend*” OR “insulin?depend*” OR “earl* diabet*” OR “auto?immun* diabet*” OR “sudden onset diabet*” OR “insulin* defic* absolut*” OR “acidos* diabet” OR “juvenil* diabet*” OR “child* diabet*” OR “keto* diabet*” OR “labil* diabet”* OR “britt* diabet*”)(title, abstract, keywords) AND (“death” OR "decease" OR “demise” OR “die” OR “dead” OR “loss of life” OR “life time” OR “lifetime” OR “life span” OR “duration of life” OR “mortality” OR “mortalit*” OR “length of life” OR “life duration” OR “longevity” OR “natural life” OR “operating life” OR “survival”) (title, abstract, keywords)
