# Supplementary material for: Diabetes duration or age at onset and mortality in insulin-dependent diabetics: a systematic review and meta-analysis
Source: Diabetol Metab Syndr. 2023 Jul 1;15:147. doi: 10.1186/s13098-023-01113-x (PMC10314605; doi:10.1186/s13098-023-01113-x)
Supplement: Supplementary file 2 — Additional file 2: Table S2. Quality assessment of observational studies included in the meta-analysis by NOS. NOS Newcastle–Ottawa scale. [file 13098_2023_1113_MOESM2_ESM.doc]

Table S1 Quality assessment of studies using Newcastle-Ottawa Scale for Cohort Studies for total mortality

| NOS scale | Modan et al(1991) | COLLADO-MESA et al(1997) | NlSHIMURA et al(1998) | Mühlhause  et al (2000) | ASAO et al(2003) | Barcel ó et al(2007) | Dawson et al(2008) | SECREST et al(2010) | Washington et al(2014) |
| --- | --- | --- | --- | --- | --- | --- | --- | --- | --- |
| A-Selection (maximum 4*) |  |  |  |  |  |  |  |  |  |
| 1.Representativeness of general community population | * | * | * | * | * | * | * | * | * |
| 2.The reference group was drawn from the same community | * | * | * | * | * | * | * | * | * |
| 3.Ascertainment the exposure of different age at onset( diagnosis) or different diabetes duration | * | * | * | * | * | * | * | * | * |
| 4.Outcome of interest was not present at baseline | * | * | * | * | * | * | * | * | * |
| B-Comparability (maximum 2*) |  |  |  |  |  |  |  |  |  |
| 5.Controlled for one variable | 0 | 0 | 0 | 0 | * | 0 | 0 | 0 | 0 |
| 6.Controlled for 2 or more variables | 0 | 0 | 0 | 0 | * | 0 | 0 | 0 | 0 |
| C-Outcome (maximum 3*) |  |  |  |  |  |  |  |  |  |
| 7. Outcome of interest was certificated by hospital or local municipal registration | * | * | * | * | * | * | * | * | * |
| 8. Adequate duration of follow-up(one year) | * | * | * | * | * | * | * | * | * |
| 9.Adequacy of follow-up rate (>90%) of cohorts | * | * | * | * | * | * | * | * | * |
| Total scores (maximum 9*) | 7 | 7 | 7 | 7 | 9 | 7 | 7 | 7 | 7 |

“*” meant the study was corresponded to the NOS criteria,” 0” meant the study wasn’t correspond to the NOS criteria

Table S1 Quality assessment of studies using Newcastle-Ottawa Scale for Cohort Studies for total mortality

| NOS scale | Gagnum et al(2015) | Marshall et al(2016) | Cheung et al(2017) | Gomes et al (2017) | Rawshani et al (2018) | Conway et al (2018) | Groop et al (2018) | Majaliwa et al (2022) |
| --- | --- | --- | --- | --- | --- | --- | --- | --- |
| A-Selection (maximum 4*) |  |  |  |  |  |  |  |  |
| 1.Representativeness of general  community population | * | * | * | * | * | * | * | * |
| 2.The reference group was drawn  from the same community | * | * | * | * | * | * | * | * |
| 3.Ascertainment the exposure of different age at onset( diagnosis) or different diabetes duration | * | * | * | * | * | * | * | * |
| 4.Outcome of interest was not present at baseline | * | * | * | * | * | * | * | * |
| B-Comparability (maximum 2*) |  |  |  |  |  |  |  |  |
| 5.Controlled for age | * | * | * | 0 | 0 | 0 | 0 | 0 |
| 6.Controlled for 2 or more variables | * | * | * | 0 | 0 | 0 | 0 | 0 |
| C-Outcome (maximum 3*) |  |  |  |  |  |  |  |  |
| 7. Outcome of interest was certificated by hospital or local municipal registration | * | * | * | * | * | * | * | * |
| 8. Adequate duration of follow-up(one year) | * | * | * | * | * | * | * | * |
| 9.Adequacy of follow-up rate (>90%) of cohorts | * | * | * | * | * | * | * | * |
| Total scores (maximum 9*) | 9 | 9 | 9 | 7 | 7 | 7 | 7 | 7 |

“*” meant the study was corresponded to the NOS criteria,” 0” meant the study wasn’t correspond to the NOS criteria

Table S1 Quality assessment of studies using Newcastle-Ottawa Scale for Case-Control study for total mortality

| NOS scale | Kostraba et al(1991) | Rendas-Baum et al (2006) |  |  |  |  |  |  |
| --- | --- | --- | --- | --- | --- | --- | --- | --- |
| A-Selection (maximum 4*) |  |  |  |  |  |  |  |  |
| 1.The case definition was adequate | * | * |  |  |  |  |  |  |
| 2.The cases were consecutive or are obviously representative series of cases | * | * |  |  |  |  |  |  |
| 3.The reference group was drawn from the same community | * | * |  |  |  |  |  |  |
| 4. No history of endpoints were present in the reference group | * | * |  |  |  |  |  |  |
| B-Comparability (maximum 2*) |  |  |  |  |  |  |  |  |
| 5.Controlled for one variable | 0 | 0 |  |  |  |  |  |  |
| 6.Controlled for 2 or more variables | 0 | 0 |  |  |  |  |  |  |
| C-Exposure (maximum 3*) |  |  |  |  |  |  |  |  |
| 7. Exposure was certificated by hospital or local municipal registration | * | * |  |  |  |  |  |  |
| 8. Same method of ascertainment for cases and controls | * | * |  |  |  |  |  |  |
| 9. Same non-response rate for both groups | * | * |  |  |  |  |  |  |
| Total scores (maximum 9*) | 7 | 7 |  |  |  |  |  |  |

“*” meant the study was corresponded to the NOS criteria,” 0” meant the study wasn’t correspond to the NOS criteria
