# Supplementary figures and images for: Diabetes duration or age at onset and mortality in insulin-dependent diabetics: a systematic review and meta-analysis
Source: Diabetol Metab Syndr. 2023 Jul 1;15:147. doi: 10.1186/s13098-023-01113-x (PMC10314605; doi:10.1186/s13098-023-01113-x)

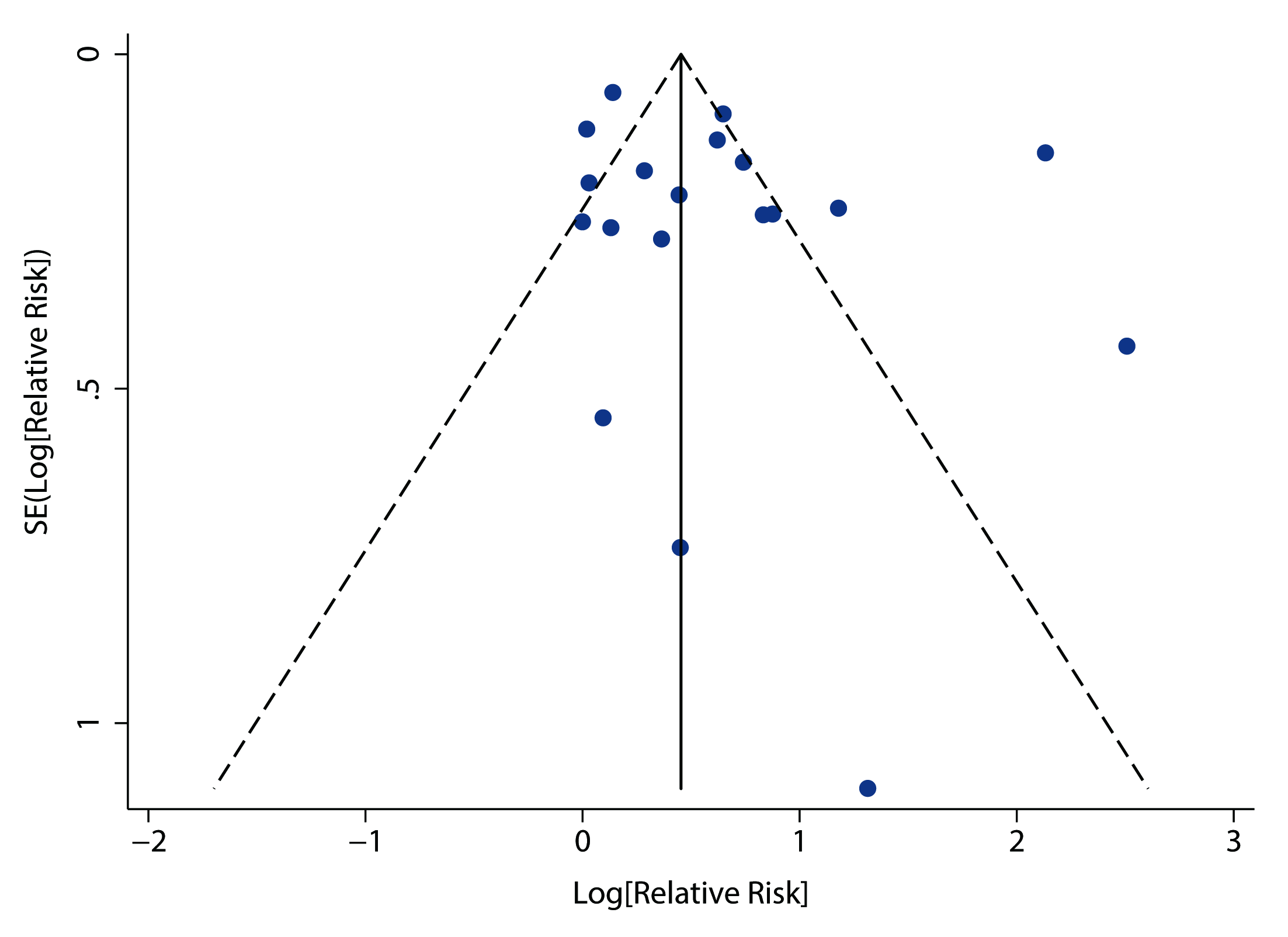

Supplement: Supplementary file 3 — Additional file 3: Fig. S1. Funnel plot for the association between age at onset and risk of total mortality in IDDM patients. IDDM insulin-dependent diabetes mellitus. [file 13098_2023_1113_MOESM3_ESM.tif]
